# Supplementary material for: Competition between physical search and a weak-to-strong transition rate-limits kinesin binding times
Source: PLoS Comput Biol. 2024 May 20;20(5):e1012158. doi: 10.1371/journal.pcbi.1012158 (PMC11142708; doi:10.1371/journal.pcbi.1012158)
Supplement: S1 Text — Additional demonstrations, investigations, and validations of computations described in the main text. (PDF) [file pcbi.1012158.s001.pdf]

S1 Text (Supporting Information) for "Competition between physical search and a weak-to-strong transition rate-limits kinesin binding times"

Trini Nguyen, Babu Janakaloti Narayanareddy, Steven P. Gross and Christopher E. Miles

May 13, 2024

Table A: Posterior Density Method Validation. Simulated data was generated using the ADP release model with known simulated values, shown in the table. A sequential Monte Carlo approximate Bayesian computational algorithm was performed on the synthetic data and recovered the simulated values, listed as estimated values in the table. The same number of simulations as data points in experiments were used.

Parameter	Simulated Value	Estimated Value
$k_{\text{off}}^{\text{ADP}}$	0.8	0.78
$k_{\text{on}}^{\text{ADP}}$	1000	1021.3
$k_{\text{off}}^{\text{ADP,Fast}}$	6.3	6.22
$k_{\text{on}}^{\text{MT}}$	10	9.83
$k_{\text{off}}^{\text{MT}}$	1	0.89
D_m	1000	987.56
κ_w	0.005	0.0057

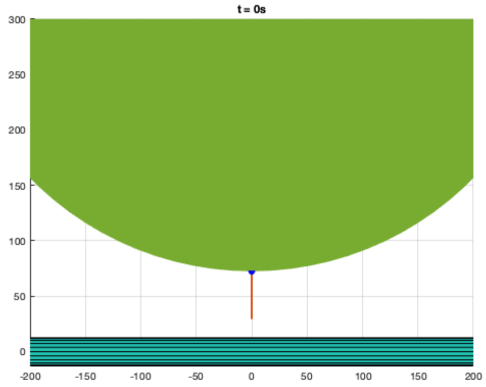

(a)

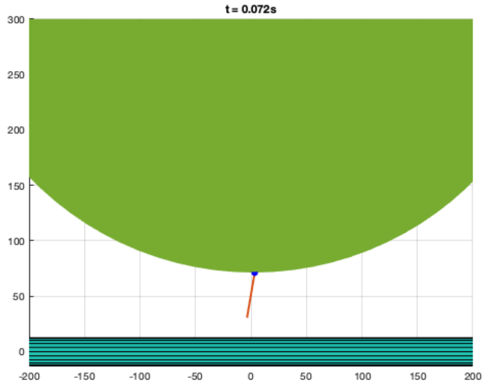

(b)

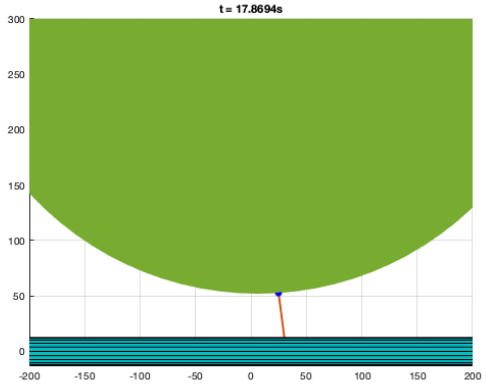

(c)

Fig A: Simulation Snapshots. The simulation starts as in (a), where the motor (red line) is anchored (blue dot) to the bottom of the cargo (green sphere). The microtubule (turquoise cylinder) is centered at (0,0), and the axes depict locations of other components with respect to the microtubule center, in nanometers. As the simulation continues, the cargo and the motor diffuses with respect to force laws (b). The simulation ends when the motor strongly binds to the microtubule (c). Time (in seconds) at which each event occurs is shown above figures.

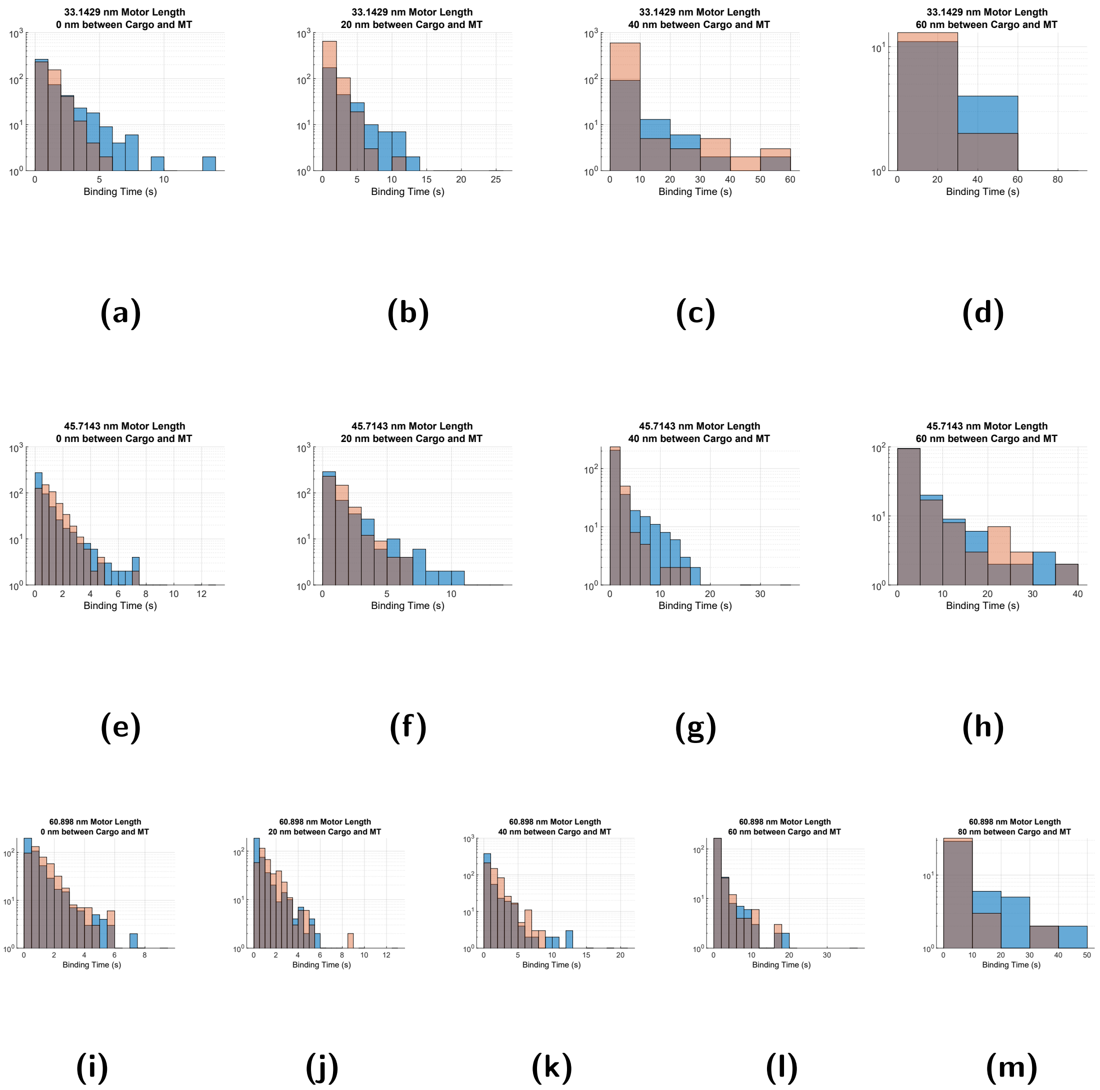

Fig B: Full distributions of binding times. Simulated data from ADP release model (red) is plotted over experimental data (blue). Overlap between distributions is shown in grey.

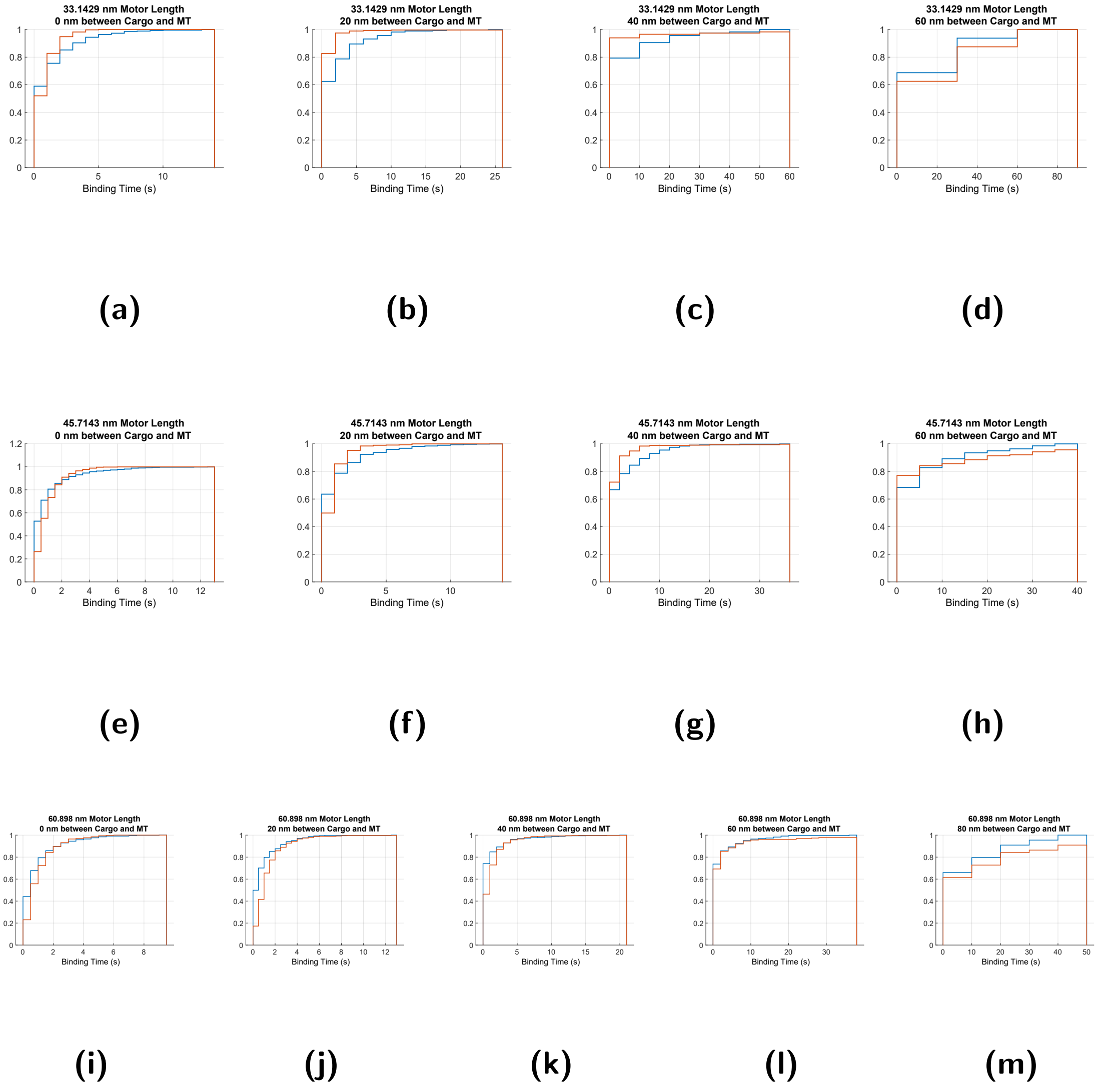

Fig C: Cumulative distributions of binding times. Simulated data from ADP release model (red) is plotted over experimental data (blue). Same information as Figure B but CDF instead of PDF.

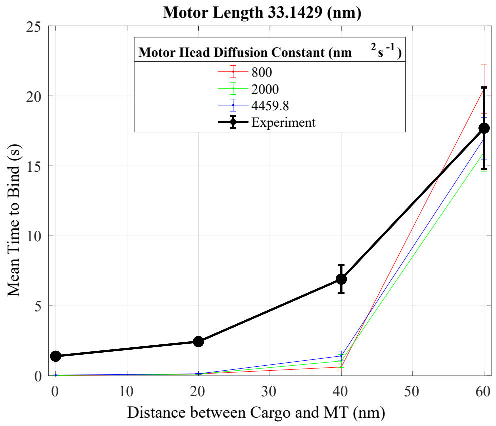

(a)

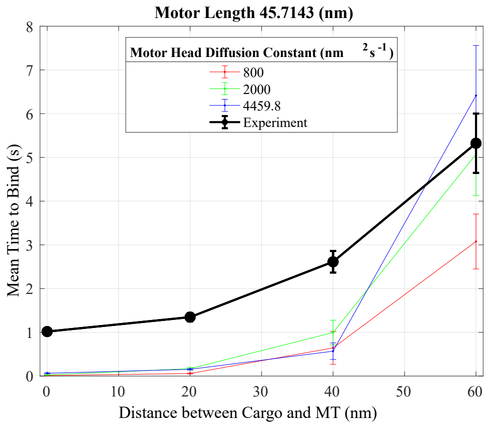

(b)

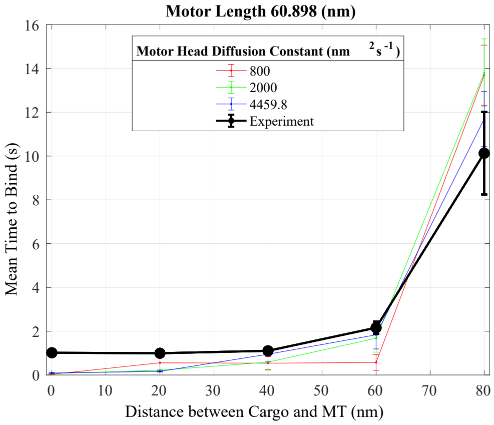

(c)

Fig D: Influence of motor head diffusion on binding time. A parameter sweep of the diffusion constant of the motor head was conducted. Data are presented as mean ± SEM.

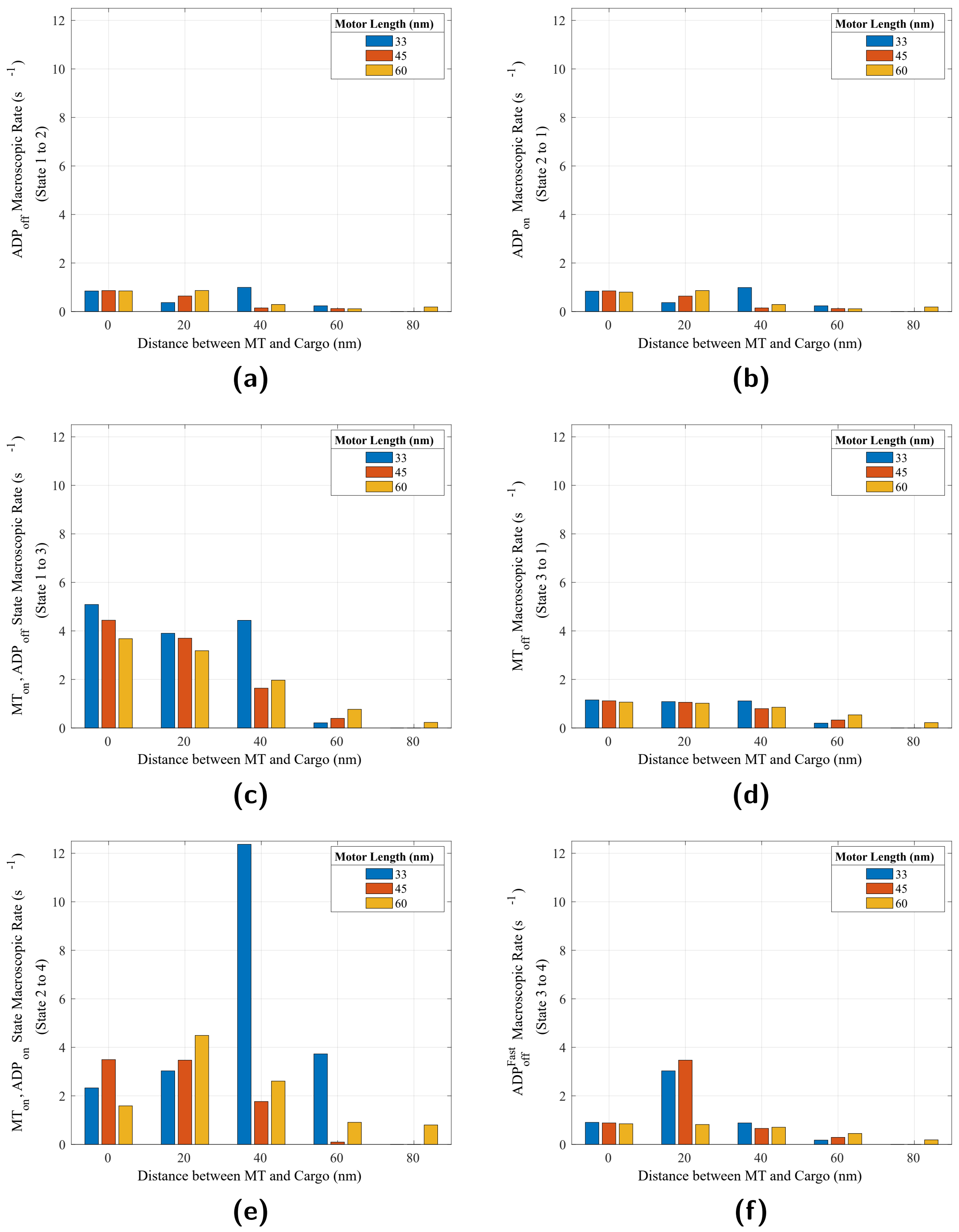

Fig E: Macroscopic rates of state transitions. Rates for each transition in the ADP+Diffusion model estimated with respect to average distance between the microtubule and the cargo for each motor length. Parameters from Table 1were used.

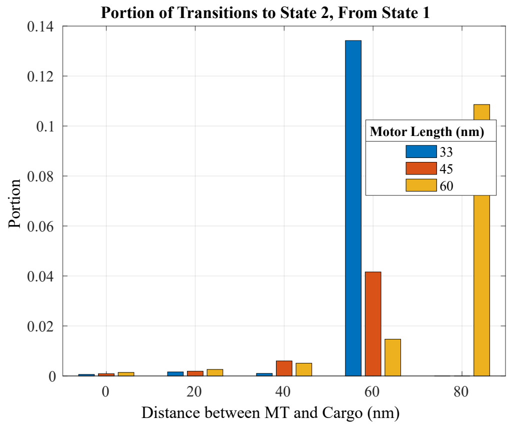

(a)

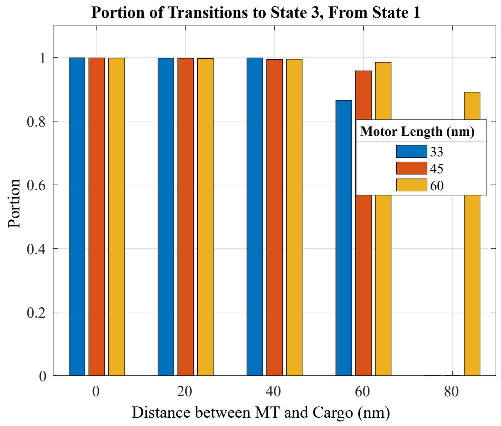

(b)

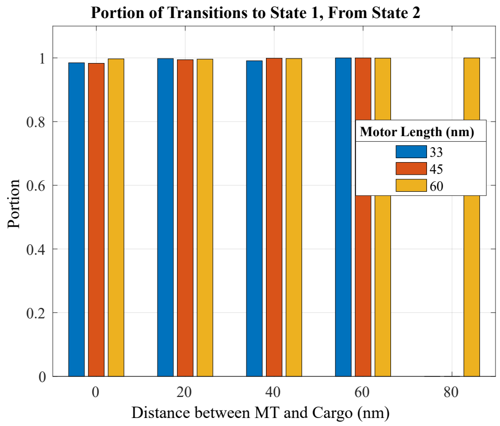

(c)

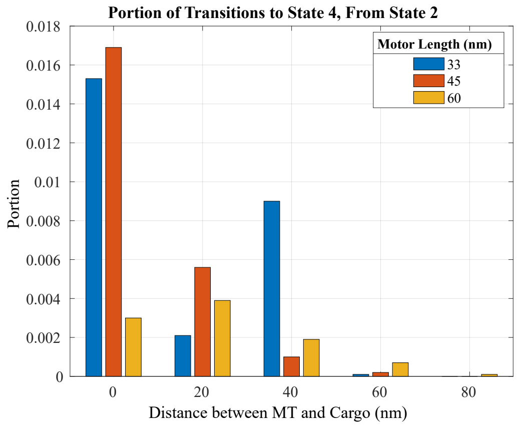

(d)

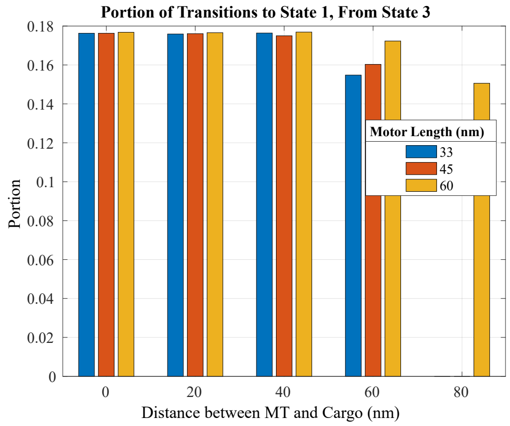

(e)

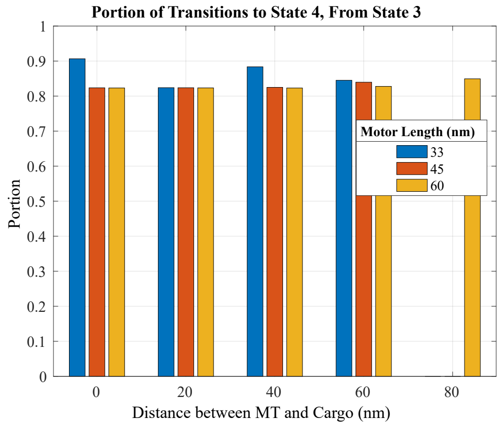

(f)

Fig F: Proportion of Transitions. Of the two possible transitions *out* of each state, the portion of transitions (or effective probability) are shown. Parameters from Table 1were used.

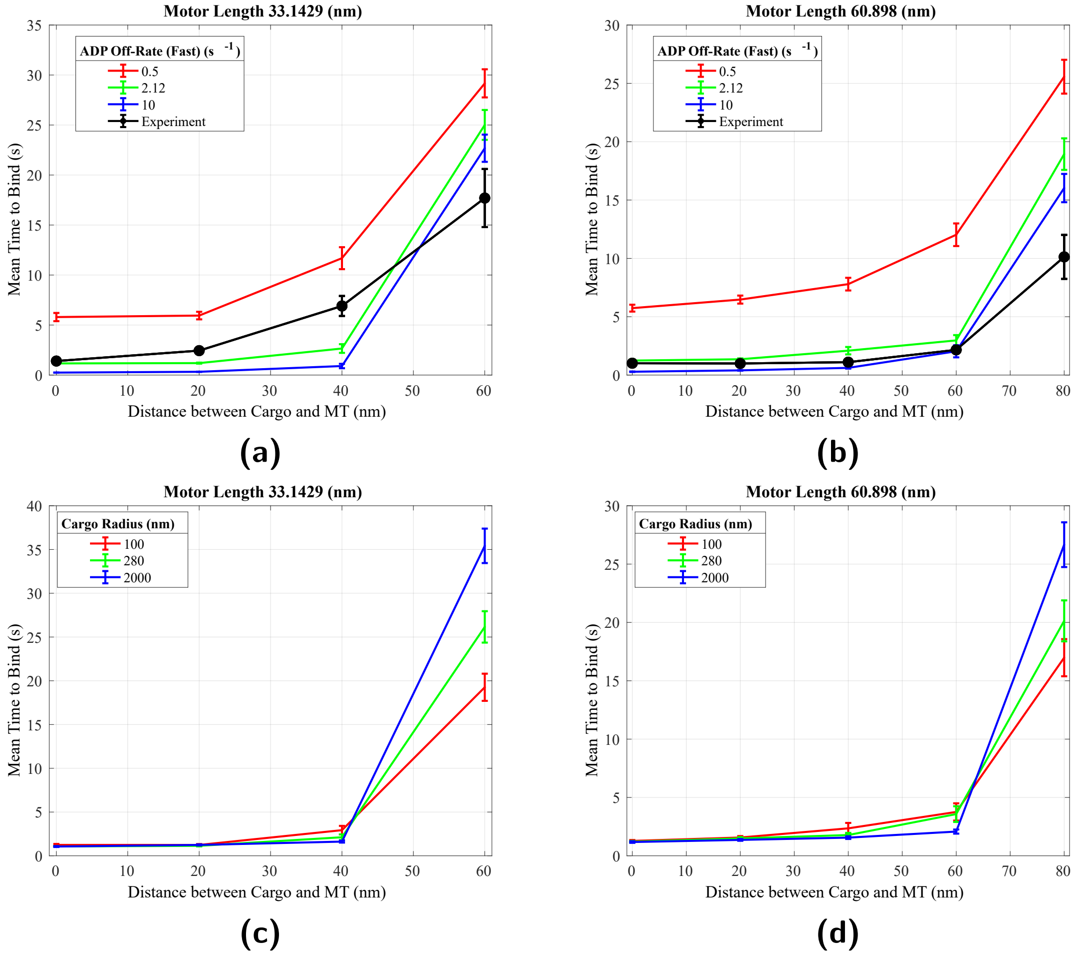

Fig G: Other Parameter Sweeps on Binding Times. $k_{\rm off}^{\rm ADP,Fast}$ (a-b) and cargo size (c-d) were varied in the ADP release model. Data are presented as mean $\pm$ SEM.

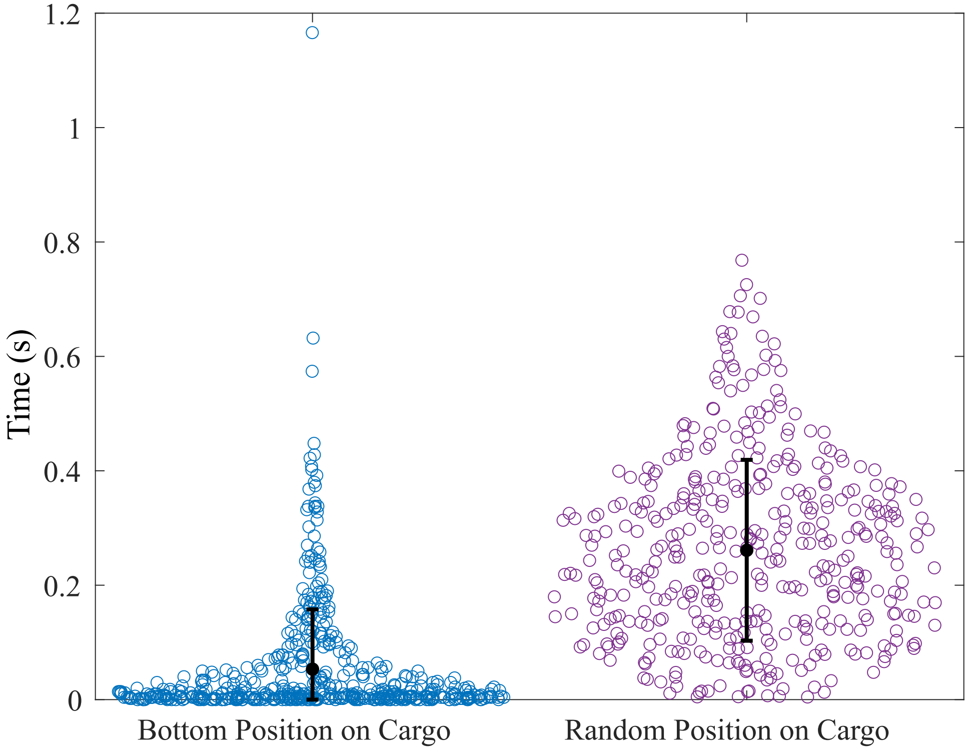

Fig H: Cargo Rotation Time. The motor anchor's initial position on the cargo is either fixed at the bottom of the cargo (left) or randomized (right), and the time for the motor to perform a diffusive search of the microtubule was simulated. $n = 500$.

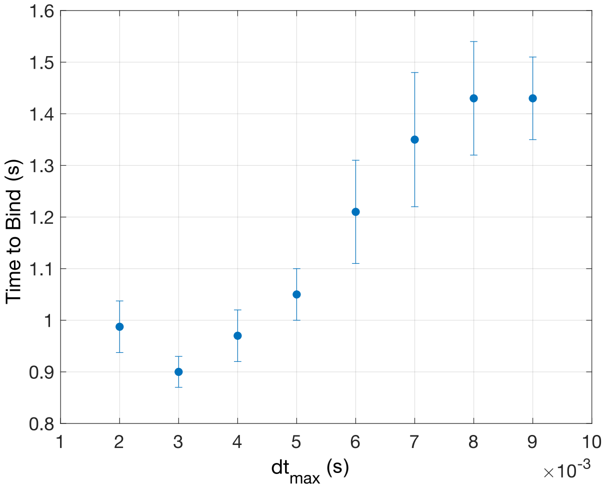

Fig I: Time step convergence study. Maximum time step was varied and converged to a common binding time. 0.004 was the largest maximum time step that resulted in a binding time that is relatively similar to the results from smaller maximum time step. $n = 1000$.

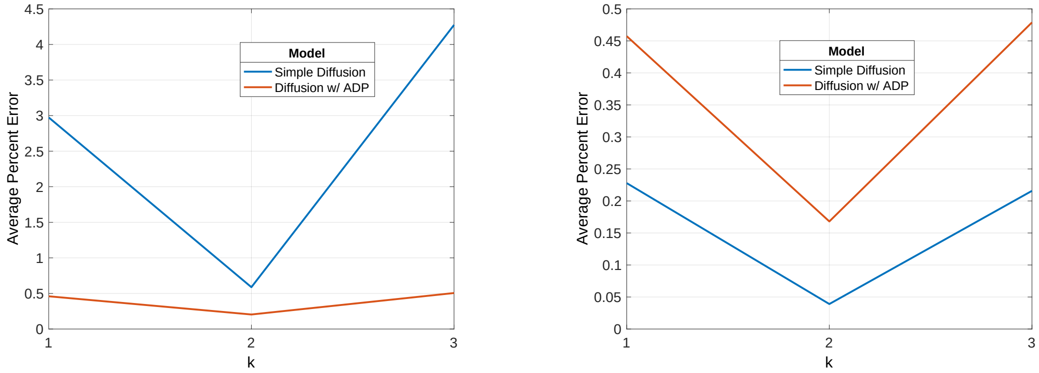

(a) Synthetic data from ADP release model **(b)** Synthetic data from Simple Diffusion model

Fig J: Cross-Validation Test. Synthetic data was simulated using either the ADP release model (a) or the Simple Diffusion model (b), and both the ADP release and the Simple Diffusion models' performance were evaluated using k-fold cross-validation.

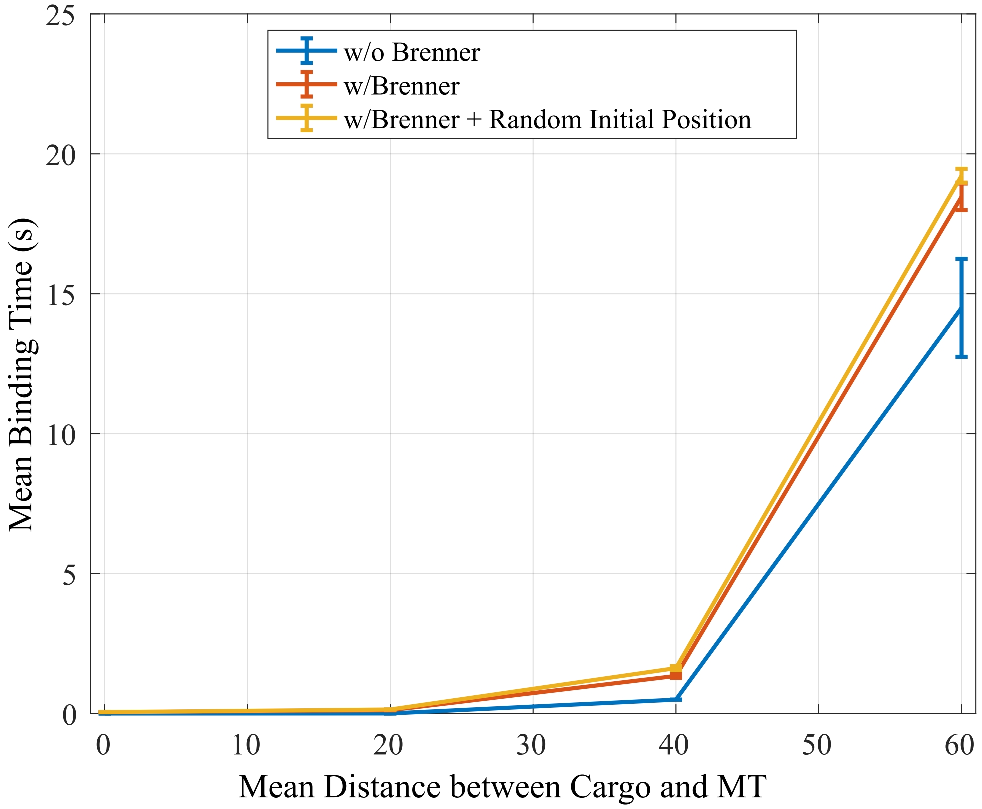

Fig K: Impact of Hydrodynamic Effects On Binding Time. To investigate the role of hydrodynamic near-wall effects, we modified the rotational and translational diffusion coefficients by their z-dependent orthogonal corrections via the classical Brenner formulae, neglecting the parallel-to-wall asymmetry as an approximation. The blue curve without correction shows the same prediction as the main text diffusion model for 33 nm motors. The red curve adds the Brenner correction, showing a slight increase in binding time at far distances. To further magnify the effect, random starting orientations (yellow) of the cargo add additional slowdown with hydrodynamics but fail to explain the short distance delays.

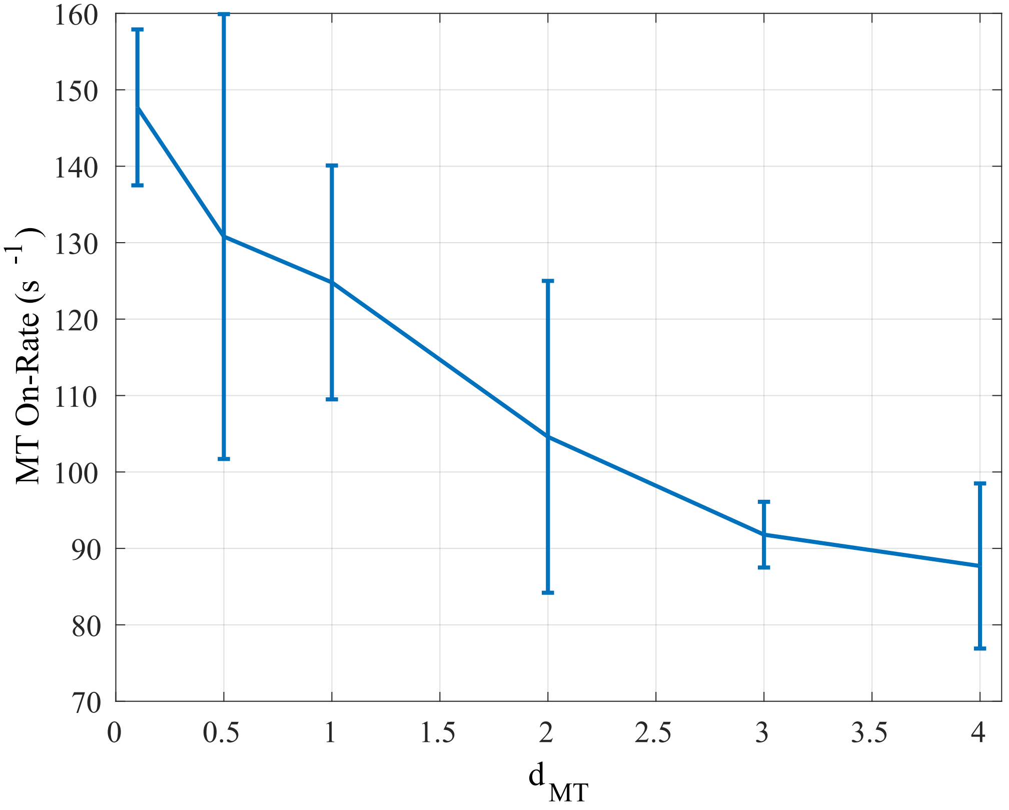

Fig L: Role of Motor-Microtubule Binding Radius in Fitting. To investigate how changing the binding radius d_{MT} affects the inferred parameter values, we sweep over ranges smaller and larger than the one considered in the main text (5 nm) and fit the full model on all 3 motor lengths. As d_{MT} becomes smaller, k_{MT} becomes larger, but remains on the same order of $\approx 100\mathrm{s}^{-1}$. Over this range, cumulative absolute fitting errors differed at most by 0.01s.
