# Supplementary material for: Competition between physical search and a weak-to-strong transition rate-limits kinesin binding times
Source: PLoS Comput Biol. 2024 May 20;20(5):e1012158. doi: 10.1371/journal.pcbi.1012158 (PMC11142708; doi:10.1371/journal.pcbi.1012158)
Supplement: Attachment — Submitted filename: ResponsesToRef.pdf [file pcbi.1012158.s002.pdf]

Reviewer #1: This is a worthy contribution that combined results from optical trapping and simulations to estimate the time it takes a kinesin motor on a spherical cargo to bind to a microtubule. Others have focused on the rotational diffusion of a cargo and how the motor itself diffuses around on the cargo. In this paper an additional rate-limiting step (ADP release from motor) is proposed.

This information, although done here for a very simplified system, could be useful to understand when and how a vesicle with motors will actually start moving. The manuscript is written well and logically organized, I found it easy to follow. I recommend publication with some changes. I have some major comments and several minor ones :-

Major Comments :-
The ADP release step seems to slow down the kinesin-microtubule binding by ~1 second when the binding is most favorable (cargo is very close to microtubule; Fig 3). What does this number (~1 second) really mean for cargo inside a cell which could have several kinesin and dynein motors close to the microtubule ? Earlier papers from the Gross group (and also from other groups) have brought up the possibility that kinesin is outnumbered by dynein motors on some cellular cargos. Given the On/Off rates for dynein, what are the implications of the present findings to a cargo which has both kinds of motors ? There should be some discussion on this aspect.

We thank the reviewer for this suggestion and agree with the assessment that we must more clearly connect our work to other contexts, e.g., collective transport. In those scenarios (for which many computational models exist), the "effective" on-rate for kinesin-1 motors that most closely reconciles data is on the order of ~1 seconds (see e.g., "Tuning multiple motor travel via single motor velocity." 2012). Our work "explains" from a microscopic perspective where this 1 second binding time arises: a mix of physical searching for the microtubule, and a delay induced by ADP release. Importantly, it also "predicts" how this rate might be modulated by physical or chemical factors, whereas previously it was entirely phenomenological. We have added discussion of this point to the introduction and discussion.

Beyond this, *in vivo* is complicated by many factors that make it challenging to even speculate how well this single-molecule experiment would predict those scenarios. In some sense, the spirit of the study is the reductionist approach, upon which further details (such as *in vivo* factors or multiple motors) should be investigated in future work.

Section "Broader Lessons for Probing Protein-Protein Interactions"
The discussion here is not really broad. It should go beyond motor-microtubule binding (considering the way the Introduction to the paper is written). I strongly feel that situations

outside this (motor-microtubule) example should be discussed. How often, and where else is an ADP-dependent rate relevant? What about prot-prot interactions where ADP is not a factor?

We have added some more concrete examples to this section.

Minor comments :-
"of diffuse importance across cellular function"
... conveys a negative outlook to the paper. What are the authors trying to say? Should diffuse be re-worded?

This sentence has now been updated.

"After this initial binding, other motors associated with the cargo attach to the filament and go through
cycles of reattachment."
Most kinesin binding rates are known from in vitro bead-kinesin assays where kinesin is very dilute and it is largely a single kinesin-to-microtubule interaction. The point that authors make is valid in a more in vivo context (many motor), but perhaps not so much to the presently known measures of binding times done in vitro. So, relevance of this statement in the context of this study is unclear.

We had originally meant this to be a broader discussion of *in vivo* motor attachment, but for the sake of clarity, we have removed this sentence.

"detectable direction of the bead"

detectable direction of the bead
OR
detectable motion of the bead ?

This sentence has now been updated.

Simple diffusion model appears closer to experimental data in Fig3C. Then why is the average percent error higher for diffusion model in Fig 3D?

ABC are results of fits to the entire data (across the three different motor lengths). D shows cross validation analysis. For comparison, the horizontal lines show the average error across the fitting seen in ABC. Caption has been updated to improve coherency.

Lines 113-114
You should mention the magnitude of this delay in the text here for the reader to appreciate better (delay looks like ~1 sec from Fig 3a)

This suggestion has been incorporated.

Reviewer #2: In this manuscript, the authors study the binding process of a kinesin motor to the microtubule. Because the binding process can be defined in many different ways, for example including diffusion of the motor with its cargo close to the microtubule or other geometric aspects, the authors choose to study a well-defined system that has been experimentally realized: a single motor attached to a bead held in an optical trap at a defined height above the microtubule. When the motor pulls the bead against the trap's restoring force, it will unbind and eventually rebind to the microtubule. This time between the unbinding and the rebinding event is conceptualized by different mechanistic models fitted to experimental data. From this modeling approach, the authors conclude that a model that includes an ADP-state dependence of the motor head is more consistent with the experimental data than a model based only on a diffusive search.

The authors use simulation-based inference methods to fit a stochastic simulation of a coarse-grained model to the experimental data. Overall, using such advanced statistical methods to combine stochastic models with experimental data is an important step forward in the field of computational biology. The presentation of the data and the logic in the text is very clear. The conclusions are solid and therefore I recommend publication in PLOS CB after the authors have addressed my comments which I suspect will improve the quality of the paper.

1) As presented in the manuscript, the bead is always kept at a specific height above the MT by a feedback trap. This scenario is of course very artificial and not very likely to occur in cells or in vitro experiments. I am wondering if it would not have been possible to switch off the feedback once the motor has been unbound to mimic the scenario of a bead starting its binding process at a defined height above the MT. I would suggest that the others could use their simulation to find out what will happen to the binding process if the bead is not fixed at a certain height.

This is an interesting suggestion but prohibitively challenging to implement. After the motor detaches from the track and "resets" into the laser trap, the only reason we know what height to start the simulations at, is because of the z-centering of the trap. Without this effect, it would be unclear what the appropriate height to start the cargo at would be (or what it would even mean to start at different ones).

We also note that controlled cargo-microtubule spacing does happen in vivo, e.g. dynactin acts as a tether to pull cargo closer, some MAPs push cargo away. More details on this scenario have now been added in the discussion section.

2) Although the authors claim that they work under single-motor conditions in their experiments, they do not show any dilution curve, therefore there is still the possibility of multi-motor conditions.

We have added further details in the methods section to clarify the single motor condition. Even more validation can be found in the 2024 Communications Biology paper focused on the experimental technique and its validation.

3) Fig.1: It's not clear why the authors labeled the fourth cartoon "walking outside trap". The motor is not walking outside the trap, it is walking away from the trap center. I don't know if the authors ever see a bead that is pulled outside of the laser beam.

This suggestion has been incorporated.

4) Fig 3: According to the text, panel C is only a validation plot. This could be indicated in the title of the plot. Is there a reason why the D+A model systematically overestimates the binding time for the 60 nm motor?

We thank the reviewer for identifying this confusing portion and have tried to re-write for clarity. Specifically, panels ABC fit all motor lengths simultaneously for one parameter set. Hence, there is an inherent tradeoff in fitting experimental data points better than others. Indeed, it is intriguing that the 60 nm fit seems to consistently overestimate, however we were unable to identify any specific reason this may be. Importantly, Panel D validation shows that even leaving out this experimental setup, the D+A model still predicts the data points with higher accuracy than just the diffusion model alone. Most interestingly, the cross-validation shows that the 33 nm motor is perhaps the most anomalous, causing the highest error when left out of the training data.

5) In the caption of Fig. 4, the authors say "approximate Bayesian Computation (ABC), but in the text, they talk about "sequential approximate Bayesian Computation (sABC).", which could be confusing. I think it would be nice if the authors could also include in Table 1 the parameter estimates from the sABC, extracted from the posteriors of Fig 4. I don't know the best practice. Maybe to report the mean or the median of the posteriors? Furthermore, it is

interesting to see that the priors for the kADP,off, and kMT,on are much more restrictive than for the other parameters, was there a reason for this?

The specific ABC method and prior selection has now been clarified in the manuscript.

6) I think the effective description of the model needs clarification. It is not clear at all what times are measured and what is shown in fig. 5A. In line 204 it says: “fraction of time stochastic simulations of the full binding process spent in State 1 and 2,...” The title of the plot says: ”Proportion of time spent in states 1 or 2”. I think the “or” should be an “and”?. Is proportion always with respect to the total binding time,? How do the authors calculate these proportions? Let’s say the mean time in state 1 is , the mean time in state 2 is , and the total time is . Is the proportion that they plot: + / or do they first determine the proportion and then average, like <(t_1 + t_2)/t_tot>? Panel C: I find it very confusing that the rates that are shown in this panel kADP,off and so on are not the same rates (numerical values) as in Fig.2 Those rates are somehow effective rates and therefore should also get different symbols.

We agree this was confusing as-written and have modified the Figure to (1) have different notation to clarify the % averaged-rates (vs. the raw rates with previously same notation).

7) Line 223 “Figand” -> “Figure”

Correction has been made.

8) I understand that the authors cannot exclude all possible models and can only show that their model is consistent with the data. This point was very well addressed in the Discussion. However, one possibility that the authors mention in line 344 is the well-known result of the distance-dependent diffusion coefficient of an object close to a surface. The authors could use the Brenner formula to estimate this effect.

Incorporating the full hydrodynamics is a complex undertaking that requires bespoke numerical codes (see work from e.g., the late Aleks Donev) outside the scope of this manuscript. However, we agree it was necessary to further investigate this beyond what was discussed in the previous submission. Therefore, we have implemented a (crude) approximation of the hydrodynamic near-wall effect to estimate its effect on the binding time. See newly add Figure S11. In these simulations, we added the Brenner-corrected rotation and diffusional perpendicular diffusion coefficients applied to all directions, under the assumption that the z-direction to the microtubule is the dominant factor in binding time. In the figure, we see that (1) if the motor is downward facing upon trap reset, there is very little effect. And (2) if the motor is randomly oriented, the effect of hydrodynamics is

larger (in slowing down the binding time) but still does not explain the order of delay observed. Moreover, we note that in a previous preprint by the Gross lab (Bovyn 2020 BioRxiv 10.1101/2020.07.13.201434v1), adjusting the bead size did indeed slightly modify the binding time, suggesting that rotations may play some, but not a significant role in the binding time.

9) I was surprised to see that the authors do not discuss a potential experiment changing the [ADP] concentration. The [ADP] concentration would change the rates in the system and thus indicate if the system is indeed influenced by the ADP state of the motor. Unfortunately, it was also not clear what the [ADP] concentration was in the experiment and if an ATP regeneration buffer was used.

We thank the reviewer for this intriguing suggestion. Yes, an [ADP] concentration experiment does seem plausible in-theory, but interpreting the impact of [ADP] concentration on binding time is prohibitively challenging. While unbound, the motor head state's competition for ATP and ADP cannot be disentangled without significantly more experiments, precisely titrating both ATP and ADP to see their relative impact on binding time. Even with these measurements, it might not be possible to say with certainty how the competition plays out (when only downstream binding can be observed) For this reason, we defer on this investigation. We have added a bit of discussion of this, including reference to the ATP buffer discussed in the 2024 Communications Biology experimentally-focused work: "Experiments were carried out at RT in motility buffer supplemented with 2 mM ATP and oxygen-scavenging system (0.25 mg ml^{-1} glucose oxidase, 30 μg ml^{-1} catalase, 4.5 mg ml^{-1} glucose)"

10) In the supplements, the authors should consistently use SI units: it is neither (secs), nor (sec), it is only (s) for units in seconds.

This has been corrected

Fig S5. The rates should get symbols and the average rates used for the results of Fig.5 should be listed somewhere.

These suggestions have been incorporated.

Reviewer #3: The authors develop a simple Brownian dynamics simulation to study the initial attachment kinetics of kinesin to the microtubule (MT). They consider two mechanisms, one where motor attachment is limited by diffusion only, versus the other where ADP release following diffusional search is rate-limiting. They find the latter to better

agree with experimental data. They make careful arguments about validation and limitations of their model. This is a potentially useful contribution to understanding the behavior of the kinesin motor that may also find applications in examining the attachment kinetics of other motors to their track. However, there are several points that need clarification and elaboration:

- Fig 3D: Average Percent Test Errors for 33nm and 60nm are over 50% for both mechanisms, which I think cannot be used to support that the better fit by the D+A model (diffusion and ADP release) is not just because it has more parameters.

We agree that this % seems quite high but we point out that, by eye, the fits are quite reasonable. Due to this non-linear fitting procedure, where classical metrics like R^2 cannot be used, we used % average error. This has the nice feature of “normalizing” the error across data points, but suffers from the issue that small differences in the short times become quite magnified. Overall, we feel that the error is honestly and transparently reported in the work.

- Line 425: Typo "K-560-His" should be "DK-560-His"

Fixed.

- It will be very helpful to define various vectors and quantities introduced in pp15-17 in a figure, either Fig 2, SI Fig 1, or in a new figure.

A table of variable names has been included.

- Line 478: Statement here indicates that their model is 3-dimensional (3D). But SI Fig 1 seems to indicate this is a 2D model. If this is indeed a 3D model, an explanation of how the orientation of the cargo (theta; line 509) is assigned and handled in Eq 2, as Langevin equation for the rotational diffusion is more involved, often requiring the use of quaternion.

The model is indeed 3-dimensional, but 2-dimensional snapshots (with primitive graphics) are shown. In the absence of hydrodynamic effects, the motion of the sphere is isotropic, so translations and rotations are easily handled by additive translations and a single rotation, as explained in the methods section.

- Clarify different versions of theta: in Eq 2, it has a tilde, on line 509, it is a scalar, and in places below, a vector symbol is used.

This is now fixed.

- How does the simulation handle cases when the cargo diffuses away from the MT?

It is stated in the methods section that only simulations where the cargo binds to the microtubule within 100 seconds are considered, directly mimicking the experimental assay.

- Table 2: They use a fixed 5nm as the MT binding range (d_MT). If d_MT is shorter, k_on^MT will decrease, making diffusional encounter to be more rate-limiting. I suggest to test a few more values of d_MT to check how sensitive their fit parameters vary (especially k_off^{ADP,fast} vs k_on^MT).

Figure S12 has been added where we investigate the effect on k_{MT} by sweeping over the parameter d_{MT}. Intuitively, smaller d_{MT} increases k_{MT}, but it remains on the order of ~100/s. Moreover, this change does not significantly change the overall quality of fit, giving a very similar overall % error (<5% difference). This makes sense since the "weak binding" is effectively a single component of the model handled by these two parameters.

- Their experimental buffer contained ATP (line 434). Explain what will happen when no ATP is present. According to their model, the initial binding should not be affected because ATP is not needed in this process.

Indeed, this observation is correct. However, without ATP, the motor would not step, and therefore its binding could not be detected in this experimental assay. Therefore, ATP is indeed necessary for investigation although not part of the proposed mechanism.
